# Status of youth access to and participation in development interventions: Data from agro-pastoral areas of east and west hararghe zones, Oromia Regional State, Ethiopia

**DOI:** 10.1016/j.dib.2023.109276

**Published:** 2023-05-29

**Authors:** Muluken G. Wordofa, Getachew S. Endris, Chanyalew S. Aweke, Jemal Y. Hassen, Jeylan W. Hussien, Dereje K. Moges, Million Sileshi, Abdulmuen M. Ibrahim, Kadija Kadiro, Kidesena Sebesibe

**Affiliations:** a School of Rural Development and Agricultural Innovation, P.O.Box 138, Haramaya University, Dire Dawa, Ethiopia; b College of Social Sciences and Humanities, Haramaya University, Ethiopia; c School of Agricultural Economics and Agribusiness, Haramaya University, Ethiopia; d Regional Manager, CAGED & EXCAM Projects at Haramaya University

**Keywords:** Agro-pastoral, Youth, Participation, Development programs, Access, Impact, Income-generating activities, Livelihoods

## Abstract

The rationale behind generating this dataset lies on the fact that there are limited data on the status of agro-pastoral youth participation in programs, projects and development interventions by the public sector, NGOs and other stakeholders. Moreover, the relationship between youth participation in interventions and changes in their livelihoods has not been properly investigated, documented and shared. Traditionally, field-based research has focused on household heads and excluded male and female youth in many contexts. The unavailability of such data severely limited the capability of various actors to make evidence-based and informed decision. It also hampered the design and implementation of youth-focused development interventions. To this end, a survey was conducted among agro-pastoral youth residing in four Woredas of East and West Hararghe Zones of Oromia Regional State, Ethiopia. A total of 398 male and female youth were randomly selected and interviewed using 12 enumerators and 5 supervisors. Participation was on a voluntary basis and informed consent was obtained from the respondents. The survey questionnaire contained information on basic socio-economic and demographic features, access to services and infrastructures, youth livelihood and income-generating activities (IGAs), and youth participation in programs, projects and development interventions, among others. The collected data were entered into a STATA software, cleaned and analysed through descriptive and inferential statistics. The outputs of the analyses were summarized in Tables, Charts and Graphs. Since the youth represent the majority of the working force in Ethiopia, they deserve a special attention. If handled properly, they can be a force for positive change. Therefore, such dataset is needed to help local level planning, implementation, monitoring and evaluation of youth livelihood transformation projects and programs. Since the data contained in this article are disaggregated by gender, Woreda and Zone, this can foster the promotion of specific projects and programs that can address expressed needs of male and female youth in agro-pastoral areas. It can also facilitate agro-ecological based implementation of development interventions. The dataset can also enable researchers, practitioners and other decision-makers to make comparative analysis on agro-pastoral youth employment, engagement in on-farm and non-/off-farm IGAs, determinants of youth participation in development programs and interventions, and impact of youth participation on livelihood transformation. The summarized dataset is provided in this article. A copy of the questionnaire is provided as a supplementary material.

**Specifications Table**

**Table:** 

Subject	- Economics, Econometrics and Finance - Social Sciences
Specific subject area	- Economic Development and Growth - Economics - Welfare - Social Science - Planning and Development
Type of data	- Table - Chart - Graph
How the data were acquired	Data were obtained through a questionnaire survey conducted in agro-pastoral context. A copy of the questionnaire is provided as a supplementary material. The questionnaire was developed by the LASER PULSE research team in close collaboration with Purdue University and local research translation partners. A total of 12 trained enumerators and 5 supervisors were used to pilot test the questionnaire and gather primary data.
Data format	- Raw data from surveys (.csv format) - Analysed with STATA - Codebook – description of variables in the dataset - Filtered by gender, Zone, and Woreda
Description of data collection	Data were collected from male and female youth (15-29 years old) located in 4 Woredas/districts of East and West Hararghe Zones. The research covered 12 Kebeles and a total of 398 youth. Roughly 50% of the sample were female youth. Selection criteria included: gender, education, economic and social status, and resource endowment.
Data source location	Institution: Haramaya University City/Town/Region: Haramaya/Oromia Regional State Country: Ethiopia
Data accessibility	Repository name: Mendeley Data Data identification number: 10.17632/85jrjvp7pm.3 Direct URL to data: https://data.mendeley.com/datasets/85jrjvp7pm/3 Processed data are included in this article

## Value of the Data

• The data presented in this article are useful because they contain a detailed account of socio-demographic characteristics of agro-pastoral youth; employment and participation in the labor market; youth engagement in on-farm and non-/off-farm income generating activities (IGAs); agricultural production, income and food security; perception of youth about agricultural employment; level of satisfaction with current occupation; access to basic services and infrastructure; and status of youth participation in programs, projects, and other development interventions.
• These datasets can be used by graduate students, researchers, local level practitioners, NGOs, and decision-makers at various hierarchies.
• Researchers can use these datasets to analyze determinants of youth participation in development interventions, evaluate the impact of participation on selected welfare and livelihood outcomes, and design research proposals. Woreda and Zonal governments and their development partners can use these datasets to inform their priority setting and resource allocation decision regarding youth livelihood transformation in agro-pastoral context. NGOs and other stakeholders can also benefit from these datasets by refocusing their program activities on male and female youth, revising their targeting criteria, and strengthening extension and advisory services, community-based organizations (CBOs) and youth and women's groups.

## 1 Objective

Ethiopia is a country in which more than 80% of the population lives in rural areas. The country has one of the highest youth populations in Africa, with approximately 49.5% of its population aged 15 to 29 years [1]. Data from the country's Central Statistical Agency (CSA) show that 67.9% of male youth aged 15 to 29 rely on the agricultural sector for their livelihoods compared to the 37.3% female youth in the same age group [2]. Pastoralists and agro-pastoralists in Ethiopia occupy approximately 61% of the country's landmass [3]. Somali region has the largest proportion of pastoralists (53%), followed by Afar (29%) and Borana (9%), and the rest 8% are found in the Gambella, Benishangul, and Tigray regions of the country [4]. Pastoralism and agro-pastoralism provide livelihoods for more than 12 million Ethiopians, who derive most of their income from keeping livestock and complement it with farming in the case of agro-pastoralists [2,5]. However, agro-pastoralists have been suffering a longstanding political and economic marginalization and the recurrent livelihood crisis, economic inefficiency, impoverishments and increasing vulnerability are partly, if not wholly, attributable to decades of economic and political marginalization. Though blessed with diverse natural, environmental, ecological, cultural and economic resources, pastoral and agro-pastoral areas of Ethiopia have been portrayed as backward and uncivilized margins of the state [6]. Thus, when compared to the country's agriculture-dominated highland areas, pastoral and agro-pastoral areas are generally ignored in terms of providing basic social services, economic infrastructure, and mechanisms that can help develop resilient adaptation to livelihood risks such as resource-based inter communal conflicts and acute food insecurity associated with natural climatic variability and human induced climate changes [7]. The purpose of this dataset is to provide an overview on the status of youth access to and participation in prevailing interventions and programs by the public sector, NGOs, and other civil society organizations.

## 2 Data Description

### 2.1 Variable Description and Measurement

The dataset contained in this data article comprises several sections. In all the sections, data were first presented for the full/pooled sample. Moreover, data were disaggregated by gender, Zone and Woreda. There were also statistical tests conducted (i.e., t-statistic/$x^{2}$-test). Table 1 provides the key variables, their definitions and levels of measurement.

**Table 1:** Key variables, their definitions and level of measurement.

Characteristics/Variable	Definition	Level of measurement
Age (years)	Age of the respondent	Number of years
Education (years)	Education level of the respondent	Number of years of schooling
Family size (no.)	Number of family members sharing the same dwelling unit	Number of family members
Gender (%)	Gender of the respondent	1 if male, 0 otherwise
Marital status (% married)	Marital status of the respondent	1 if married, 0 otherwise
Access to employment	Proportion of youth currently employed	1 if yes, 0 otherwise
Non-/off-farm IGAs	Proportion of youth participating in non-/off-farm income-generating activities (IGAs)	1 if participant, 0 otherwise
Current occupation	Primary occupation of the respondent	1 if agriculture/farming, 0 otherwise
Employment status	Employment status of the respondent	1 if self-employed, 0 otherwise
Current job/IGA was my childhood dream	Proportion of youth having the same job as their childhood dream	1 if yes, 0 otherwise
Interest in starting own business	Proportion of youth with an interest in starting own business	1 if yes, 0 otherwise
Experience in farming (years)	Number of years of experience in agriculture	Number of years
Land holding size (ha)	Land holding size of the respondents	Hectare (ha)
Land registration certificate (%)	Proportion of respondents having a land registration certificate	1 if yes, 0 otherwise
Livestock possession (TLU)	Total number of livestock possessed	Tropical Livestock Unit (TLU)
Expenditure (ETB/year)	Expenditure for productive assets per year	Ethiopian Birr (ETB)
On-farm income (ETB/year)	Total annual farm income obtained from selling crop and livestock products	ETB
Household Dietary Diversity Score (HDDS)	Household Dietary Diversity Score computed following the methodology indicated in [8,^9^,10]	Score
Food Consumption Score (FCS)	Food Consumption Score computed following the methodology indicated in [11]	Score
Youth's ownership of asset	Proportion of youth owning asset	1 if yes, 0 otherwise
Youth control over use of income	Proportion of youth having control over income	1 if yes, 0 otherwise
Youth access to and decision about credit	Proportion of youth with access to and decision about credit	1 if yes, 0 otherwise
Extension/advisory services	Proportion of youth with access to extension/advisory services	1 if yes, 0 otherwise
Farmers’ Field Schools (FFSs)	Proportion of youth with access to farmers’ field schools	1 if yes, 0 otherwise
Farmers’ Training Centers (FTCs)	Proportion of youth with access to farmers’ training centers	1 if yes, 0 otherwise
Pastoral Training Centers (PTCs)	Proportion of youth with access to pastoral training centers	1 if yes, 0 otherwise
Received training (last 5 years)	Proportion of youth who received training in the last five years	1 if yes, 0 otherwise
Received on-the-job training	Proportion of youth who received on-the-job training	1 if yes, 0 otherwise
Access to credit and saving MFI	Proportion of youth with access to credit and saving microfinance institutions (MFIs)	1 if yes, 0 otherwise
Participation in SME promotion/activities	Proportion of youth participating in small and medium enterprise (SME) promotion activities	1 if yes, 0 otherwise
Membership in CBOs	Proportion of youth who are members of community-based organizations (CBOs)	1 if yes, 0 otherwise
Community welfare group	Proportion of youth who belong to a community welfare group	1 if yes, 0 otherwise
Religious group	Proportion of youth belonging to a religious group	1 if yes, 0 otherwise
Networks/viable platforms	Proportion of youth with access to networks and viable platforms	1 if yes, 0 otherwise
Women's group	Proportion of youth participating in women's group	1 if yes, 0 otherwise
Youth group	Proportion of youth participating in youth group	1 if yes, 0 otherwise
Women and children affairs office	Proportion of youth participating in women and children affairs office	1 if yes, 0 otherwise
Access to PSNP	Proportion of youth with access to productive safety net program (PSNP)	1 if yes, 0 otherwise
Irrigation-based agricultural production (irrigation coops)	Proportion of youth participating in irrigation cooperatives	1 if yes, 0 otherwise
Primary cooperatives	Proportion of youth participating in primary cooperatives	1 if yes, 0 otherwise
Cooperative union	Proportion of youth participating in cooperative union	1 if yes, 0 otherwise
Catholic Relief	Proportion of youth with access to Catholic Relief services	1 if yes, 0 otherwise
CARE Ethiopia	Proportion of youth with access to CARE Ethiopia services	1 if yes, 0 otherwise
Other NGOs (emergency relief, LLRP and others)	Proportion of youth with access to other NGOs, including lowland livelihood resilience project (LLRP)	1 if yes, 0 otherwise
NGOs total	Proportion of youth participating in the activities of NGOs	1 if yes, 0 otherwise

### 2.2 Basic Socio-Demographic Characteristics of Respondents

**Fig. 1:**
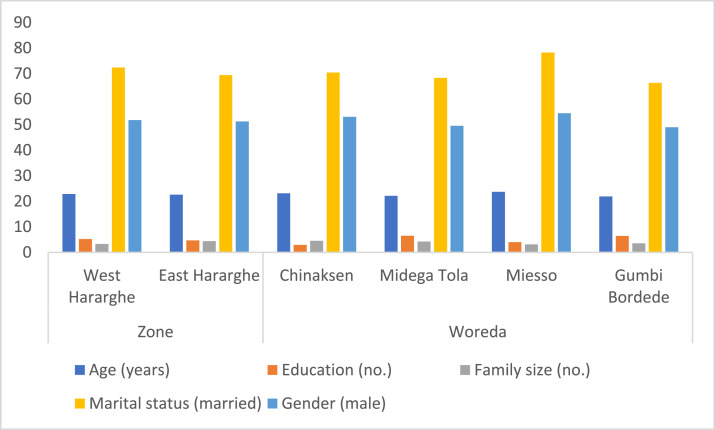
Zonal and Woreda level comparisons of basic characteristics of study participants.

Data on socio-demographic characteristics were presented in Table 2 (for the full sample, male and female youth) and Fig. 1 (for the two Zones and four Woredas). As can be seen in Table 2, the average age of the respondents was 23 years. They also possess a lower level of educational qualification, i.e., grade five, on average. About 71% of them were married with an average family size of four individuals. Early marriage is a characteristic feature in agro-pastoral areas.

**Table 2:** Basic socio-demographic characteristics of respondents.

Characteristics/Variable	Pooled Sample (*n* = 398)	Female Youth (*n* = 193)	Male Youth (*n* = 205)	t-statistic/$x^{2}$-test
Age (years)	22.67 (0.20)	22.15 (0.31)	23.16 (0.25)	-2.51 ***
Education (years)	4.96 (3.96)	3.89 (0.27)	5.98 (0.27)	-5.43***
Family size (no.)	3.82 (0.11)	3.89 (0.17)	3.76 (0.16)	0.58
Gender (%)		48.49	51.51	
Marital status (% married)	70.85	67.88	73.66	23.23 ***

The comparison between male and female youth sub-samples revealed the existence of a statistically significant difference in terms of age, educational attainment, and marital status (Table 2). More specifically, male youth were found to have better education and most of them were married compared to the female youth.

At zonal level, except for average family size, which is larger in East Hararghe (4.4) compared to West Hararghe (3.3), there was no significant difference for the other variables. The Woreda level comparison also showed a slight variability. Compared to the other Woredas covered by the study, a greater number of educated youth were found in Midega Tola and Gumbi Bordede. Likewise, although a large number of youth were married in Miesso and Chinaksen, bigger family sizes are found in Chinaksen and Midega Tola. The data did show any significant difference across the Woredas for the remaining variables (Fig. 1).

### 2.3 Youth Employment and Participation in the Labour Market

**Fig. 2:**
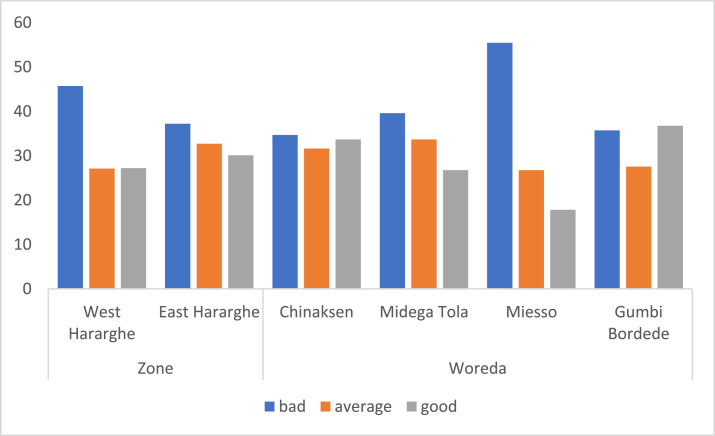
Zonal and Woreda level comparison on availability of appropriate job opportunities for youth (%).

Youth employment and participation in the labour market has been assessed using ‘availability of appropriate job opportunities’ and ‘youth engagement in on-farm and non-/off-farm income-generating activities.’ A Five-Point Likert Scale questionnaire (‘very good’ to ‘very bad’) was employed to gather data on availability of appropriate job opportunities for the full sample, male and female youth categories (Table 3). At Zonal and Woreda level, however, a Three-Point Likert Scale was used (‘bad’ to ‘good’) and the data were presented in Fig. 2.

**Table 3:** Availability of appropriate job opportunities.

Availability of appropriate job opportunity	Pooled Sample	Female Youth	Male Youth	$x^{2}$-test
Very good	7.29	5.70	8.78	3.59
Good	21.36	21.24	21.46	
Average/Neutral	29.90	27.98	31.71	
Bad	23.62	24.35	22.93	
Very bad	17.84	20.73	15.12

Table 3 shows that about 29%, 30% and 41% of the study participants rated the prospect of appropriate job opportunities as good, average and bad, respectively. This result did not vary significantly between male and female youth study participants.

At Zonal and Woreda level, however, availability of appropriate job opportunities for the youth appears to vary (Fig. 2). Many youth in West Hararghe (46%) indicated the prospect of job opportunities as ‘bad’ compared to 37% of the youth in East Hararghe who indicated the same. At the Woreda level, many youth in Miesso (55%) and Midega Tola (40%) stated that job opportunities are ‘bad’.

**Fig. 3:**
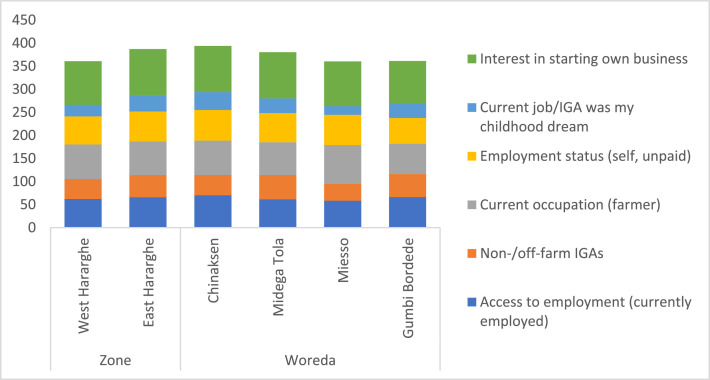
Zonal and Woreda level comparison of youth employment (%).

The engagement of youth in on-farm and non-/off-farm income-generating activities (IGAs) was given in Table 4 (for the pooled sample, male and female youth) and Fig. 3 (for Zonal and Woreda comparisons). In addition to access to employment and status of employment, this section also presents data on childhood job aspirations and interest of the youth to start their own businesses and IGAs. Regarding current occupation, the data on current youth employment status shows that about 64% of the youth are currently employed. Most of them (74%) are engaged in farming/agriculture. Youth engagement in non-/off-farm income-generating activities (IGAs) stands at 46%, with a greater proportion of male youth engaged in agriculture compared to their female counterparts. Most of the employed youth (63%) operate family farms (unpaid job, but sharing agricultural outputs). The data further shows that only about 30% of the youth (engaged in agriculture) indicated that this was their childhood dream. For most of the youth, however, this is not the case. About 97% of the respondents showed interest in starting their own business/income-generating activity (Table 4).

**Table 4:** Youth employment – on-fam and non-/off-farm (%).

Characteristics/Variable	Pooled Sample	Female Youth	Male Youth	$x^{2}$-test/t-statistic
Access to employment (currently employed)	64.07	61.14	66.83	1.40
Non-/off-farm IGAs	45.73	53.89	38.05	10.05 ***
Current occupation (agriculture/farming)	73.87	65.80	81.46	26.06***
Employment status (self, unpaid)	62.81	60.62	64.88	0.77
Current job/IGA was my childhood dream	30.15	34.72	25.85	3.71*
Interest in starting own business	97.24	95.85	98.54	2.66

The comparison between male and female youth shows significant differences in relation to access to and participation in off-/non-farm income-generating activities (IGAs) and engagement in the agricultural sector as the main occupation. The data indicates that a greater number of male youth are engaged in agriculture, while a greater number of female youth rely on non-/off-farm employment opportunities (Table 4).

At the Zonal level, the data did not show any significant difference in terms of percentage of youth currently employed between East Hararghe (66%) and West Hararghe (62%). The same is true for youth engagement in non-/off-farm IGAs (43% for West Hararghe, 48% for East Hararghe). However, the study indicates significant differences between the two zones in relation to current occupation and interest in starting own business (Fig. 3).

The Woreda level comparison also shows the existence of significant differences in terms of current occupation and interest to start own IGA (Fig. 3). Overall, most of the youth in the study area want to engage in an entrepreneurial venture by creating new jobs for themselves and other youth in their community. This ambition, if supported by relevant stakeholders, can be a means to promote self-employment and youth engagement in the economy.

### 2.4 Agricultural Production, Income and Food Security

In the above section, we presented and discussed data on youth employment in on-farm and non-/off-farm IGAs. In this section, we present additional data related to agricultural employment and outcome indicators. As indicated in Table 5, youth participants in the agricultural sector had a small land holding size (1.33 ha) and an average farming experience of five years. About 67% of the land holdings have a land registration certificate. In terms of livestock possession, the youth had an average of 3.5 tropical livestock units (TLU). Comparing male and female youth for these parameters, the data did not show any statistically significant differences. However, male youth were slightly better-off, except for TLU, which is greater for female youth.

**Table 5:** Agricultural production, income and food security (mean values; standard deviations in parenthesis).

	Pooled Sample	Female Youth	Male Youth	$x^{2}$-test/t-statistic
Experience in farming (years)	5.37 (0.23)	5.18 (0.34)	5.56 (0.30)	-0.83
Land holding size (ha)	1.33 (0.05)	1.30 (0.07)	1.35 (0.08)	-0.51
Land registration certificate (%)	67.09	66.32	67.80	0.10
Livestock possession (TLU)	3.50 (0.20)	3.63 (0.29)	3.38 (0.27)	0.63
Expenditure (ETB/year)	7,450.19 (542.83)	7,404.18 (904.22)	7,493.52 (623.56)	-0.08
On-farm income (ETB/year)	27,162.72 (2,700.06)	30,481.94 (5,036.19)	24,037.80 (2,229.80)	1.19
Household Dietary Diversity Score (HDDS)	5.37 (0.09)	5.62 (0.13)	5.13 (0.13)	2.72 ***
Food Consumption Score (FCS)	44.50 (0.97)	46.16 (1.44)	42.94 (1.31)	1.66 **

Note: ETB = Ethiopian Birr (official currency).

In terms of agricultural production, the result indicates that the youth obtained low level of farm income (i.e., ETB 27,163/year on average). The data on expenditure for productive assets and gross farm income shows no significant variation between male and female youth. However, the estimates for Household Dietary Diversity Score (HDDS) and Food Consumption Score (FCS) appear to significantly vary between male and female youth (Table 5). The findings suggest that female youth are better-off in terms of HDDS and FCS compared to their male counterparts.

**Fig. 4:**
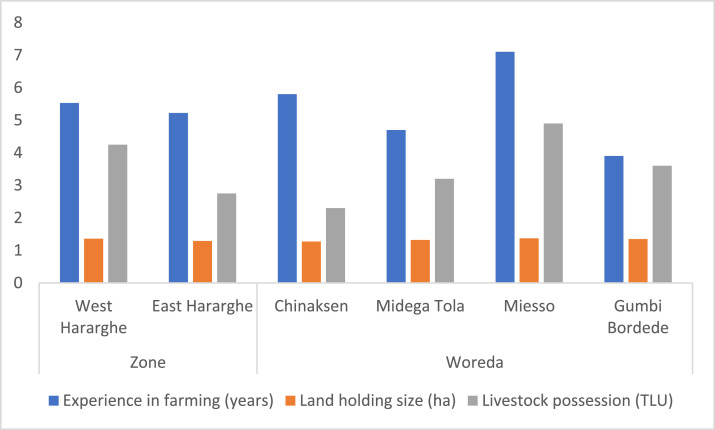
Zonal and Woreda level comparison of experience in farming, land holding size and livestock holdings.

**Fig. 5:**
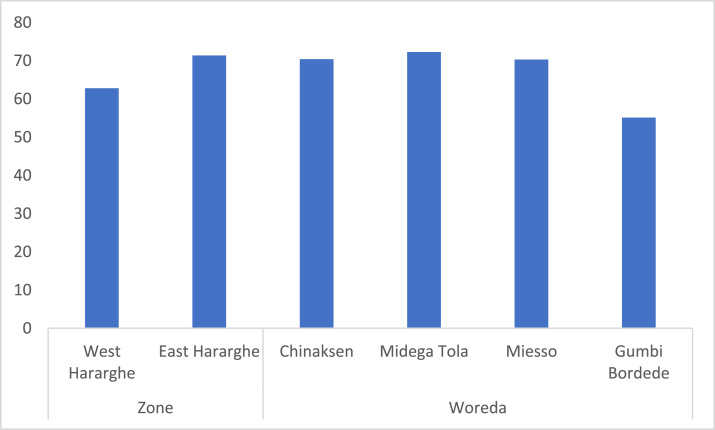
Zonal and Woreda level status of land registration (%).

**Fig. 6:**
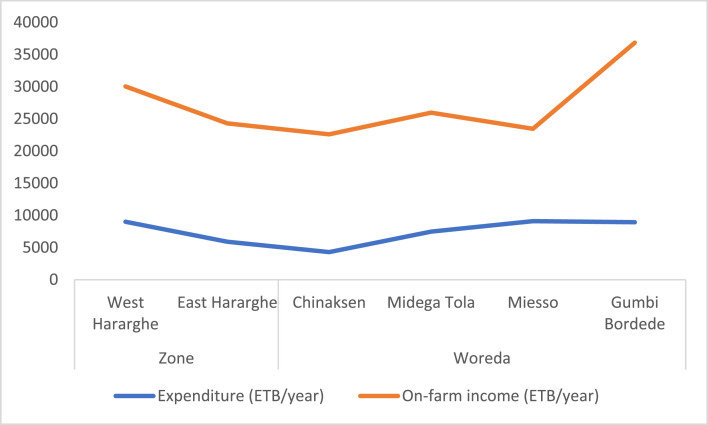
Zonal and Woreda level comparisons of expenditure and farm income.

**Fig. 7:**
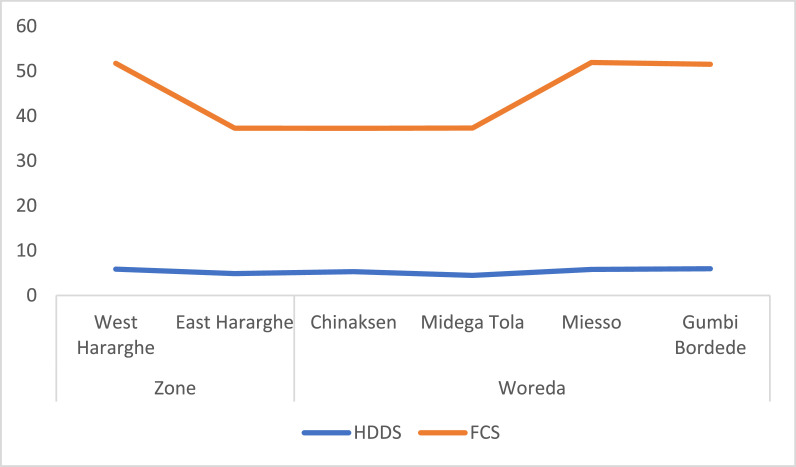
Zonal and Woreda level comparison of HDDS and FCS.

At Zonal level, there are significant differences regarding livestock possession measured in TLU (Fig. 4), land certification (Fig. 5), expenditure for productive inputs (Fig. 6), and HDDS and FCS (Fig. 7).

The data shows that youth in West Hararghe, compared to those in East Hararghe, possess more livestock, have higher HDDS and FCS, and spend more on productive inputs. In terms of land registration, however, greater proportion of youth in East Hararghe have a land certificate.

Comparing the four study Woredas, the data shows that more youth experienced in agriculture are found in Chinaksen (Fig. 4); the largest TLU is associated with youth in Miesso (Fig. 4); and more land is certified in Midega Tola (Fig. 5).

Likewise, Fig. 6 depicts Zonal and Woreda level comparative data on expenditures for productive assets and farm income. In relation to agricultural production and food security, the largest input expenditures are associated with farms located in Miesso, and the largest farm income is recorded among farms in Gumbi Bordede (Fig. 6).

Regarding food security, data on household dietary diversity score (HDDS) and food consumption score (FCS) were presented in Fig. 7. Whereas the largest FCS (51.9) are associated with youth in Miesso, the largest HDDS (5.9) are found in Gumbi Bordede (Fig. 7).

### 2.5 Youth Perception about Agricultural Employment

**Fig. 8:**
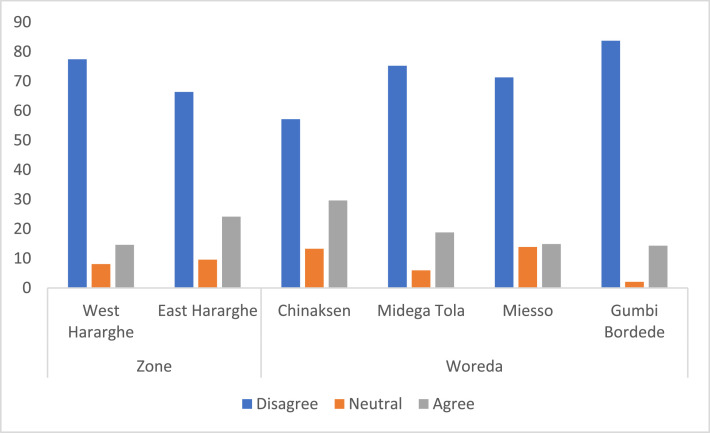
Zonal and Woreda level comparison on whether agriculture can be a basic means of livelihood (%).

Contained in this data article were also perception of youth about agricultural employment. Whereas data about the pooled sample and male and youth categories was presented using a Five-Point Likert Scale (‘strongly agree’ to ‘strongly disagree’), the Zonal and Woreda level comparisons were given using a Three-Point Likert Scale. Youth perception on whether agriculture can be a basic means of livelihood is given in Table 6 and Fig. 8. Accordingly, about 72% of the youth have the perception that agricultural sector cannot fulfil their basic livelihood necessity. Furthermore, there exists a statistically significant difference in perception between male and female youth – greater proportion of male youth (76%) believe that the agricultural sector cannot support their livelihoods adequately compared to the 67% female youth who indicated the same.

**Table 6:** Level of agreement if agriculture can be a basic means of livelihood for the youth (%).

Level of agreement	Pooled Sample	Female Youth	Male Youth	$x^{2}$-test
Strongly agree	0.75	1.04	0.49	8.82*
Agree	18.59	19.69	17.56	
Neutral	8.79	11.92	5.85	
Disagree	34.67	36.27	33.17	
Strongly disagree	37.19	31.09	42.93

At Zonal level, although the score for youth in West Hararghe is higher compared to those in East Hararghe, the difference is not significant. The Woreda level data, however, shows that there are some significant differences – greater number of youth in Gumbi Bordede indicated that they do not believe agriculture to be a basic means of livelihood (Fig. 8).

Likewise, the youth were asked about their perception on whether agriculture can be a viable profession with a reasonable financial return. The data are depicted in Table 7. Accordingly, 74% of the youth indicated that agriculture cannot be a viable profession with a reasonable economic gain. Although there was a difference between male youth (69%) and female youth (79%), the overall $x^{2}$-test shows that the difference is not significant.

**Table 7:** Level of agreement if agriculture can be a viable profession with a reasonable financial return (%).

Level of agreement	Pooled Sample	Female Youth	Male Youth	$x^{2}$-test
Strongly agree	0.75	1.04	0.49	5.95
Agree	16.83	19.17	14.63	
Neutral	8.29	10.36	6.34	
Disagree	34.92	35.23	34.63	
Strongly disagree	39.20	34.20	43.90	

**Fig. 9:**
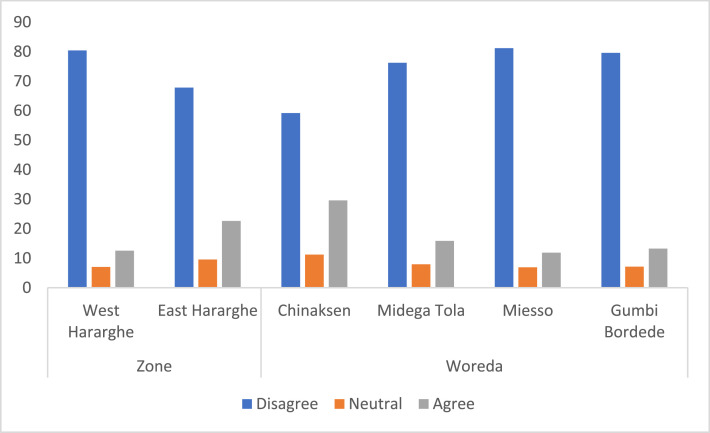
Zonal and Woreda level comparisons of youth perception on whether agriculture can be a viable profession with a reasonable financial return (%).

Looking at the Zonal level data (Fig. 9), there were significant differences, with greater proportions of youth in West Hararghe indicating that agriculture cannot be a viable profession. Similarly, at Woreda level, there exists significant difference in perception: greater proportion of youth in Miesso (81%) indicated that agriculture cannot be a worthwhile profession compared to only 59% of the youth who indicated the same in Chinaksen.

Data on youth's level of satisfaction with their current (agricultural) job is given in Table 8. About 65% of the youth indicated that they are not satisfied with their current job. Greater proportion of male youth (71%) are dissatisfied compared to female youth (58%) and the difference is significant.

**Table 8:** Level of satisfaction with current job (%).

Level of satisfaction	Pooled Sample	Female Youth	Male Youth	$x^{2}$-test
Very satisfied	3.27	5.18	1.46	12.05 **
Satisfied	18.84	23.83	14.15	
Average/Neutral	13.07	12.95	13.17	
Not satisfied	32.91	28.50	37.07	
Not very satisfied	31.91	29.53	34.15	

**Fig. 10:**
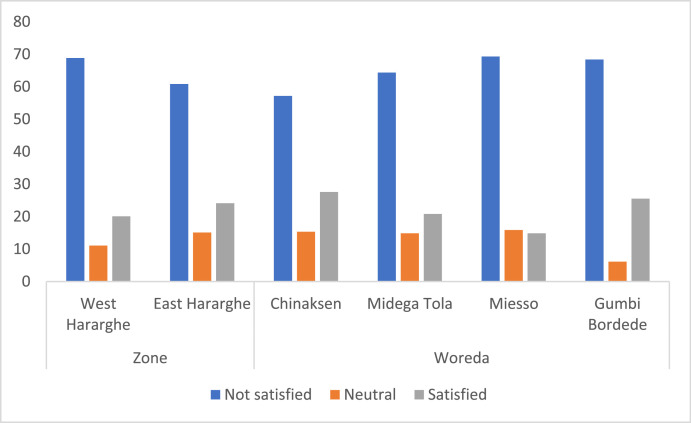
Zonal and Woreda level comparison of level of satisfaction with current job (%).

At Zonal level, although greater proportion of youth in West Hararghe were dissatisfied (69%) compared to East Hararghe (61%), the overall difference is not significant. At Woreda level, however, there are significant differences: greater proportion of youth in Miesso were dissatisfied (69%) compared to those in Chinaksen (57%). Fig. 10 presents detailed data on the Zonal and Woreda level comparisons.

### 2.6 Youth Access to Basic Services, Infrastructure and Facilities

**Fig. 11:**
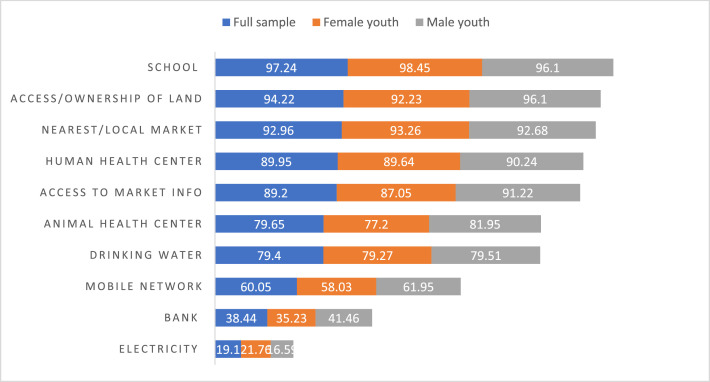
Proportion of male and female youth with access to basic services, infrastructure and facilities in selected Woredas of East and West Hararghe Zones of Oromia regional state, Ethiopia (%).

This data article also contains data on youth access to basic services, infrastructure and facilities, including school, land, market, health centers, water, telecom, banking and electricity (Fig. 11). The data refer to the gendered access to school, land, local market and market information, health center (human and animal), drinking water, telephone and electricity, and banking services. As indicated in Fig. 11, except for access/ownership of land, there was not any significant difference between male and female youth.

**Fig. 12:**
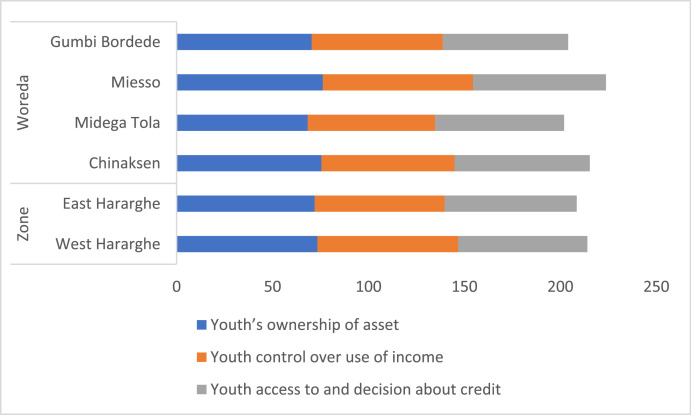
Zonal and Woreda level comparison on youth's ownership of asset, control over use of income, and decision about credit (%).

Youth ownership of asset, control over use of income, and decision about credit is presented in Table 9 and Fig. 12. The data in Table 9 shows that there are differences between male and female youth in relation to ownership of asset, control over user of income, and decision-making about credit. Female youth have less ownership of asset and decision-making right about credit. However, they are relatively better-off in relation to control over use of income (Table 9).

**Table 9:** Youth ownership of asset, control over use of income, and decision about credit (%).

Characteristics/Variable	Pooled Sample	Female Youth	Male Youth	$x^{2}$-test
Youth's ownership of asset	72.61	64.77	80.00	11.60 ***
Youth control over use of income	70.60	62.69	78.05	11.29 ***
Youth access to and decision about credit	68.09	59.59	76.10	12.47 ***

The Zonal and Woreda level data on youth's ownership of asset, control over use of income, and decision about credit is given in Fig. 12. Overall, there was no significant difference between the two zones and across the four Woredas.

### 2.7 Youth Participation in Public Extension and Advisory Services

**Fig. 13:**
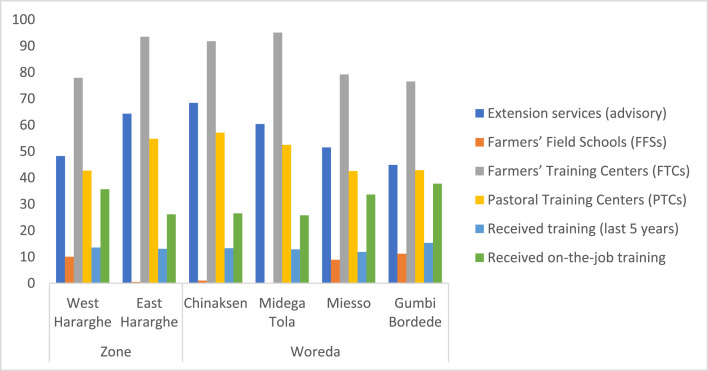
Zonal and Woreda level comparison of youth participation in extension services, FFSs, FTCs, PTCs and training (%).

An important dimension of this data article presents data on youth participation in programs, projects, networks/groups, and other development interventions of the public sector and NGOs. Data on youth participation in public extension and advisory services, including farmer field schools (FFSs), farmer training centers (FTCs), and pastoral training centers (PTCs) is presented in Table 10 and Fig. 13.

**Table 10:** Youth participation in public extension and advisory services (%).

	Pooled Sample	Female Youth	Male Youth	$x^{2}$-test
Extension/advisory services	56.28	48.70	63.41	8.74 ***
Farmers’ Field Schools (FFSs)	5.28	4.15	6.34	0.96
Farmers’ Training Centers (FTCs)	85.68	82.38	88.78	3.32 *
Pastoral Training Centers (PTCs)	48.74	40.41	56.59	10.40 ***
Received training (last 5 years)	13.32	8.81	17.56	6.60 **
Received on-the-job training	30.90	27.98	33.66	1.50

Youth participation in public agricultural extension services has been assessed to understand whether the youth involve in and benefit from the prevailing public agricultural extension and advisory system in agropastoral areas. To this end, the youth were asked to state whether they interacted with extension agents/development agents and received advisory and training services. Moreover, the youth were asked whether they participated in the activities of Farmers’ Training Centers (FTCs) and Farmer Field Schools (FFSs), including agricultural training, field days and demonstrations. The data on these parameters are given in Table 10. Accordingly, majority of the youth (56%) have received advisory services from development agents. Although 86% of the youth reported to have access to the FTCs, only 13% of them received agricultural training in the last five years. Youth participation in the FFSs is also found to be very limited. The comparison between male and female youth indicates that greater proportions of male youth participate in the extension/advisory services, FTCs, PTCs and training compared to the female youth (Table 10).

The Zonal level data shows the existence of significant differences in relation to youth participation in extension services, FFSs, FTCs, PTCs and on-the-job training (Fig. 13). More specifically, the data shows that youth participation in extension services, access to FTCs, and participation in FTCs (PTCs) is better in East Hararghe compared to West Hararghe. Youth participation in the FFSs and on-the-job training is better in West Hararghe than East Hararghe. There was no significant difference between the two zones in terms of youth participation in training. Similarly, the Woreda level aggregated data reveals significant differences regarding youth involvement in extension services, FFSs and FTCs (Fig. 13) – greater proportion of youth participate in advisory services in Chinaksen (68%) and higher proportion of youth involve in the activities of FTCs in Midega Tola (95%). However, the data did not show any significant difference across the Woredas in terms of youth participation in PTCs and training.

### 2.8 Youth Participation in MFI and SME Promotion Activities

**Fig. 14:**
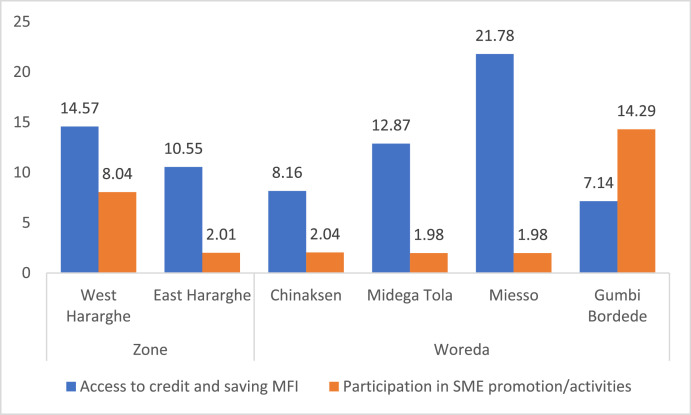
Zonal and Woreda level comparison of youth access to MFI and participation in SME promotion activities (%).

Data on youth participation in microfinance institutions (MFI) and small and medium enterprise (SME) promotion activities is depicted in Table 11 and Fig. 14. The data on the status of youth access to and participation in credit and saving Micro-Finance Institutions (MFI) shows a very low level of youth involvement (about 13%). More worrisome is their participation in Small and Medium Enterprise (SME) promotion activities of their respective Woreda (i.e., only 5%). The data indicates that this challenge is common across male and female youth in the study areas (Table 11).

**Table 11:** Youth participation in MFI and SME promotion activities (%).

	Pooled Sample	Female Youth	Male Youth	$x^{2}$-test
Access to credit and saving MFI	12.56	13.47	11.71	0.28
Participation in SME promotion/activities	5.03	4.66	5.37	0.10

The zonal level data shows the existence of significant differences between East and West Hararghe in terms of youth participation in SME promotion activities, with West Hararghe performing relatively better than East Hararghe. Overall, however, the level of youth participation in SME promotion activities is very low. In terms of participation in credit from MFI, although West Hararghe appears to be better, there was no significant difference across the two zones.

The Woreda level data indicates significant differences across the four Woredas (Fig. 14). For instance, whereas greater proportion of youth in Miesso have access to MFI credit, more number of youth in Gumbi Bordede participate in SME activities of their Woreda.

### 2.9 Youth Participation in Community-Based Organizations (CBOs), Networks and Local Groups

The data on youth participation in various CBOs, networks and local development groups are presented in Table 12. The data indicates that the youth are better represented in CBOs and community welfare groups. However, there exists significant difference between male and female youth only in relation to participation in women's group and youth group. The data did not show any significant difference regarding membership in CBOs, participation in community welfare groups, religious groups, networks/viable platforms and women and children affairs (Table 12).

**Table 12:** Youth participation in CBOs, networks and groups (%).

	Pooled Sample	Female Youth	Male Youth	$x^{2}$-test
Membership in CBOs	67.84	66.84	68.78	0.17
Community welfare group	69.85	72.54	67.32	1.29
Religious group	7.29	8.29	6.34	0.56
Networks/viable platforms	29.90	29.53	30.24	0.02
Women's group	13.07	20.21	6.34	16.83 ***
Youth group	13.32	9.84	16.84	3.91 **
Women and children affairs office	15.08	15.03	15.12	0.001

**Fig. 15:**
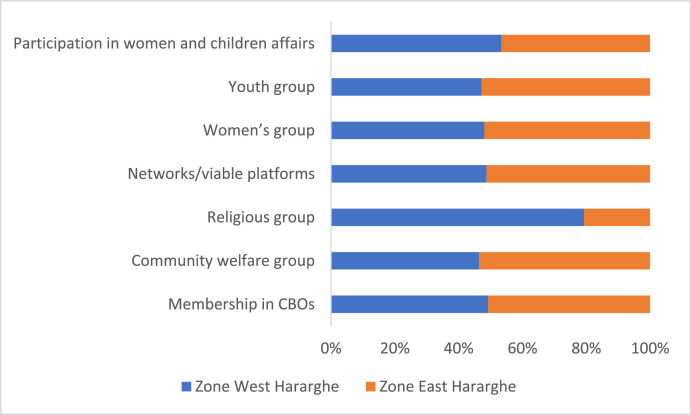
Zonal level comparison of youth participation in various CBOs, networks and local groups (%).

At zonal level, the data shows significant differences in relation to community welfare groups and religious groups (Fig. 15). Whereas East Hararghe had better youth participation in community welfare groups, West Hararghe is better in terms of youth participation in religious groups.

**Fig. 16:**
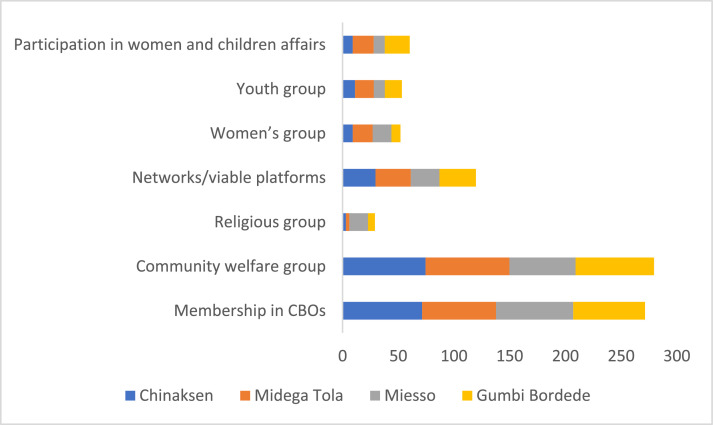
Woreda level comparison of youth participation in various CBOs, networks and local groups (%).

The Woreda level comparative data also reveals the existence of significant differences regarding participation in community welfare groups, religious groups, women's groups, and women and children affairs (Fig. 16). Midega Tola and Chinaksen are better in youth participation in community welfare group; greater proportion of youth are members of religious groups in Miesso; many youth participate in the activities of women and children affairs office in Gumbi Bordede.

### 2.10 Youth Participation in the Productive Safety Net Program (PSNP) and Cooperatives

The data on youth participation in the PSNP indicates a low level of participation (24%), with female youth participating more compared to their male counterparts. The level of youth participation in irrigation cooperatives, primary cooperatives, and cooperative unions is also found to be very low (Table 13). The data did not show any significant difference between male and female youth participation in cooperative organizations.

**Table 13:** Youth participation in the PSNP and cooperatives (%).

	Pooled Sample	Female Youth	Male Youth	$x^{2}$-test
Access to PSNP	24.12	29.53	19.02	6.0**
Irrigation-based agricultural production (irrigation coops)	2.51	2.59	2.44	0.01
Primary cooperatives	4.77	4.15	5.37	0.33
Cooperative union	9.80	8.29	11.22	0.97

**Fig. 17:**
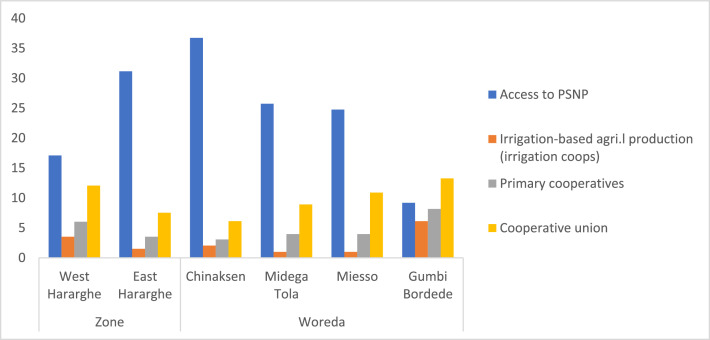
Zonal and Woreda level comparisons of youth participation in the PSNP and cooperatives (%).

The zonal level data shows the existence of significant differences regarding youth participation in the PSNP only (i.e., East Hararghe better than West Hararghe). However, at Woreda level, there exists significant differences in terms of youth participation in the PSNP and irrigation cooperatives (Fig. 17) – whereas Chinaksen is better in the PSNP, Gumbi Bordede has the largest proportion of youth participating in irrigation cooperatives.

### 2.11 Youth Participation in the Operations of NGOs

Youth participation in various NGOs has been assessed and the data shows it to be very low. Overall, the proportion of youth access to and participation in NGOs and their programs (including donor organizations, CARE Ethiopia, emergency relief, LLRP, and Catholic Relief) is 31%, with male youth participating better compared to female youth. Looking into the details of the data, youth participation in emergency relief, Lowlands Livelihood Resilience Project (LLRP) and other NGOs stands at 16%, with male youth participating more compared to their female counterparts. However, the data did not show any significant difference between male and female youth regarding participation in the activities of Catholic Relief and CARE Ethiopia (Table 14).

**Table 14:** Youth participation in the activities of NGOs (%).

	Pooled Sample	Female Youth	Male Youth	$x^{2}$-test
Catholic Relief	4.02	4.66	3.41	0.40
CARE Ethiopia	5.78	5.18	6.34	0.25
Other NGOs (emergency relief, LLRP and others)	16.33	11.92	20.49	5.34**
NGOs total^a^	30.90	25.39	36.10	5.34**

a This refers to access to/participation in donor organizations, CARE Ethiopia, emergency relief, Lowlands Livelihood Resilience Project (LLRP), Catholic Relief and other NGOs).

**Fig. 18:**
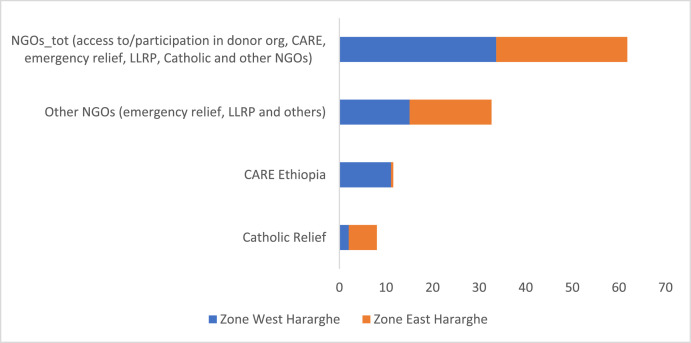
Zonal level comparison of youth participation in the activities of various NGOs (%). Source: own illustration from survey data, 2022.

At zonal level, the data did not indicate any significant difference, except for participation in Catholic Relief and CARE Ethiopia (Fig. 18) – whereas greater proportion of youth participate in Catholic Relief in East Hararghe, more number of youth participate in CARE Ethiopia in West Hararghe.

**Fig. 19:**
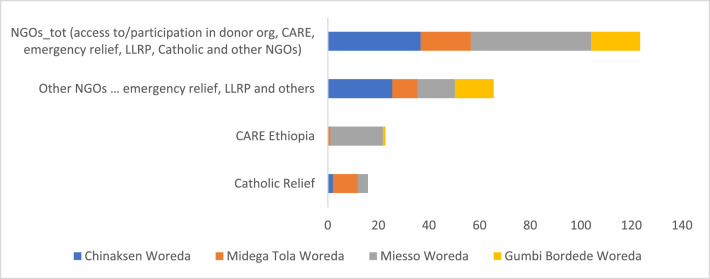
Woreda level comparison of youth participation in the activities of NGOs (%). Source: own illustration from survey data, 2022.

At Woreda level, the data shows a statistically significant difference in terms of youth participation in the activities of NGOs, including Catholic Relief and CARE Ethiopia (Fig. 19) – a greater proportion of youth participate in NGOs in Miesso (48%) and in Chinaksen (37%) compared to the other Woredas.

## 3 Experimental Design, Materials and Methods

In Ethiopia, more than 80 percent of the population lives in rural areas. The fast-growing youth population, dwindling landholding size, and growing unemployment are significant challenges the country has faced at the moment. The fact that approximately 49.5% of its population is aged 15 to 29 years makes Ethiopia one of the highest youth populations in Africa [1]. This dataset was a result of a project implemented in agro-pastoral areas of East and West Hararghe Zones, Oromia Regional State, Ethiopia. The Zones are largely affected by land degradation, frequent droughts, and the prevalence of social and resource-based conflicts leading to poverty and food insecurity [12]. The targeting of both East and West Hararghe was done to increase the scope and widen the applicability of the data to other agro-pastoral areas in Ethiopia. It was also intended to further strengthen the preparation of youth strategy document and policymaking in an agro-pastoral context.

### 3.1 Study Design and Population

The data gathering process employed a cross-sectional research design following the mixed-methods approach. This means that it combined qualitative and quantitative data generation and analysis procedures. The Positive Youth Development (PYD) [13,14] approach was also followed to guide the research process and identify youth and women's assets, agencies, contributions, and enabling environment to enhance youth livelihood transformation.

The source population are male and female youth (aged 15 to 29 years) in East and West Hararghe Zones of East Hararghe, Oromia Regional State, Ethiopia. The study population are rural youth in selected Woredas of East Hararghe Zone (Chinaksen and Midega Tola Woredas) as well as West Hararghe Zone (Miesso and Gumbi Bordede Woredas).

### 3.2 Sampling Procedures, Inclusion/Exclusion Criteria

To ensure adequate inclusion and representation of female youth respondents, there was a plan to include about 50% women/female youth. A detailed inclusion and exclusion criteria (considering gender, education, disability, refugee status, economic and social status, resource endowment) was also established to select different categories of male and female youth in the study area.

Identification of target areas and participants for the research and intervention was undertaken considering existing diversities in socioeconomic and institutional factors. Thus, the intervention Woredas/Districts, Kebeles and agro-pastoral youths were selected in consultation with the local research translation partners and other relevant stakeholders. A total of four Woredas were purposively selected from East and West Hararghe Zones (i.e., two Woredas from each zone) based on the prevalence of agro-pastoralism as a livelihood strategy, proportion of youth and women in the total population, and representatively to the rest of the Woredas in the zones. In each Woreda, three representative Kebeles (i.e., the smallest administrative units in Ethiopia) were purposively selected on the basis of agro-ecological zones, possibilities for comparisons, vulnerability context and existence/lack of development interventions by the public sector, NGOs and other organizations. Finally, a total of 398 youth and women were identified from a fresh list of residents developed for the purpose of the study and randomly selected.

### 3.3 Data Collection and Analysis

The data were obtained from primary sources using semi-structured interview schedule. The questionnaire was developed through a joint effort between the research team at Haramaya University and collaborators from the Long-term Assistance and Services for Research Partners for University-Led Solutions Engine (LASER PULSE) program at Purdue University. We designed the questionnaire based on the results obtained from a desk review conducted in the study area prior to the field-based primary data collection. This result enabled us identify key variables to be included in the questionnaire to get detailed information about agro-pastoral youth, opportunities available, status of participation in development interventions implemented by various organizations, and other socio-economic, institutional and demographic characteristics. We also consulted our local research translation partners working in the study area to suggest important elements affecting youth participation in programs, projects and interventions. Moreover, we made a reference to similar studies conducted elsewhere in agro-pastoral settings and included relevant items.

Primary data were collected by employing research assistants and local supervisors. A total of 12 research assistants/enumerators and 5 supervisors were provided an orientation training before they embark on pretesting the data collection instruments. The research assistants were selected based on their educational qualification, experience in socio-economic research, language competence, and experience in living/working in the study areas. The supervisors were instrumental in overseeing the process of data collection, together with the core research team, and provided feedback and technical support to the data collectors.

Data collection process started by pretesting all the data collection tools with selected non-project participant respondents. Following the feedback gathered through this process, the data collection instruments were refined and adjusted for the main field survey and data collection. Through the pilot testing, we ensured that the data collection instruments contained questions that were unambiguous and unbiased. A range of data quality assurance mechanisms were implemented during the field-based data collection, analysis and reporting. To ensure the collection of high-quality data, the research team employed research assistants/enumerators with the required educational background and experience in survey and qualitative data collection tools. There was a continuous supervision by the research team and local supervisors tasked with follow up and provision of on-the-spot feedback to enumerators. There was also a daily debriefing session where enumerators shared their experiences and encounters and got support from the technical team. Quantitative data was checked regularly for completeness and consistency. Food Consumption Score (FCS) was measured using both the types of food groups consumed and the frequency of consumption of these food groups, and computed by employing the procedures indicated in the World Food Program (WFP) Technical Annex 2012 [10]. In order to capture the dietary habit of the sample households, seven days recall period was used which further reduces the risk of selection bias [11]. Frequency of consumption and weights attached to each food group are used for computing food consumption score [11]. The Household Dietary Diversity Score (HDDS) is a measure of food adequacy indicating the number of food groups consumed at household level, which is considered to be an indicator for economic ability of households [8]. Dietary diversity refers to the number of food groups (e.g., cereals, vegetables, milk, meat, legumes, eggs and fruits) consumed over 24 hours recall period [9]. The HDDS score ranges from 1 to 12. The minimum is consuming one food group over the reference period and the maximum is consuming twelve food groups [8,10].

The data was partially analysed through descriptive (mean, frequency, and standard deviation) and inferential statistics (t-test, $x^{2}$-test). In all these processes, the research team, enumerators and supervisors practiced the highest level of scientific integrity and ethical procedures in gathering, processing, and analysing data.

## Ethics Statements

The entire research was conducted following the highest ethical and standard operating procedures (SOPs) of safeguarding the rights, safety and wellbeing of all human subjects participating in the research. This project will pose minimal health risk on human subjects participating in the research activities since it does not involve the collection of blood, urine or other samples directly from the respondent. Nonetheless, the research team secured ethical clearance letter (Ref. No.: IHRERC/061/2022, Date: April 05/2022) stating the same, and practiced informed consent during data collection, safeguard privacy and anonymity, exercise caution in data access, use and sharing, and ensure the overall ethical acceptability/integrity of the research.

## CRediT authorship contribution statement

**Muluken G. Wordofa:** Conceptualization, Methodology, Software, Formal analysis, Visualization, Data curation, Writing – original draft, Writing – review & editing, Funding acquisition. **Getachew S. Endris:** Conceptualization, Methodology, Investigation, Supervision, Data curation, Writing – original draft, Writing – review & editing, Funding acquisition. **Chanyalew S. Aweke:** Conceptualization, Methodology, Investigation, Supervision, Data curation, Writing – original draft, Writing – review & editing, Funding acquisition. **Jemal Y. Hassen:** Conceptualization, Resources, Supervision, Project administration, Funding acquisition, Writing – original draft, Writing – review & editing. **Jeylan W. Hussien:** Conceptualization, Resources, Supervision, Writing – review & editing. **Dereje K. Moges:** Investigation, Supervision. **Million Sileshi:** Conceptualization, Validation, Investigation, Supervision. **Abdulmuen M. Ibrahim:** Conceptualization, Validation, Investigation, Supervision. **Kadija Kadiro:** Validation, Investigation, Supervision. **Kidesena Sebesibe:** Validation, Investigation, Supervision.

## Declaration of Competing Interest

The authors declare that they have no known competing financial interests or personal relationships that could have appeared to influence the work reported in this data article.

## Data Availability

Dataset on Agro-Pastoral Youth Participation in Development Interventions in East and West Hararghe Zones, Oromia Regional State, Ethiopia (Original data) (Mendeley Data).

## Appendix Supplementary materials

Image, application 1
