# Supplementary material for: Status of youth access to and participation in development interventions: Data from agro-pastoral areas of east and west hararghe zones, Oromia Regional State, Ethiopia
Source: Data Brief. 2023 May 29;48:109276. doi: 10.1016/j.dib.2023.109276 (PMC10294086; doi:10.1016/j.dib.2023.109276)
Supplement: Supplementary file 1 [file mmc1.pdf]

Survey Questionnaire for LASER PULSE Project – English

The survey is aimed at assessing the existing nature of development interventions in terms of engaging youth and occupational aspirations of male and female youth in agro-pastoral areas of eastern Ethiopia. It also intends to identify competitive advantages and potential sources of sustainable livelihood opportunities for agro-pastoral youth. This tool is developed to gather primary data from respondents in the target territories. The data collected will only be used for research purpose and will remain confidential. The research team requests your kind cooperation and patience in providing accurate and reliable responses for the questions. You indicate your voluntary consent by participating in this interview, may we begin?

Thank you for your kind co-operation.

Information about the respondent

1.	Zone	
2.	Woreda	
3.	Kebele	
4.	Locality/village	
5.	Name of respondent	
6.	Household ID	

**Information about the Enumerator & supervisor/Assignment record**	
7. Name of Enumerator	
8. Date of interview (dd/mm/yyyy)	/ / 2022
9. Name of supervisor	
10. Duration of interview	Starting time …………… ending time …………..

Module 1: Demographic characteristics of the respondent

**Questions**	**Responses**
Sex of the respondent	1. Male ☐<br>2. Female ☐
Age of the respondent (in years)	
Number of years of formal education/schooling	
Marital status	1. Single ☐ 2. Married ☐<br>3. Divorced ☐ 4. Widowed/er ☐<br>5. Separated ☐
Your main occupation(Code b)	
**(Code b)-**Farming=1, Civil servant =2, Wage Labour =3, Business=4, Any other (Specify)=5	
Family Size and Structures	
Total number of family members (including yourself)	

Are you responsible for **day-to-day decision-making** in the **household**?
1. Yes 2. No

Module 2: Socio-economic and institutional characteristics of respondents

2.1.Do your family or yourself own land? 1. Yes 2. No
2.2.Do you or your family have a land registration certificate? 1. Yes 2. No
If yes, land holding (including rented-in/out as well as shared cropped in/out) and use (please indicate here your family's land and crops produced)

Plots	Ownership*	Size (ha)
Agricultural (crop)		
Pasture/grazing		
Residential unit		
Trees/shrubs		
Natural vegetation		
Any other (please specify)		

**1. Own 2. Rented–in 3. Share cropped 4. Rented–out 5. Any other* ________________________
2.3.How many years of experience in farming (crop, livestock) do you have?__________
2.4.What are the major crops grown in the last production season (if any)?

a. Sorghum
b. Maize
c. Wheat
d. Vegetables
e. Fruits
f. Chat
g. Any other (specify) …

Type of crop/tree/forest product	Land allocation	Total amount produced (yield) (kg)	Total amount sold (kg)	Total amount consumed at hh level (kg)	Total amount stored, given as gift (kg)	Market price (birr/kg)
Sorghum						
Maize						
Wheat						
Chat						

Do you have access to market information system? 1. Yes 2. No
2.12 Do you have livestock? 1. Yes 2. No

Livestock ownership and marketing for the household in general during the last 12 months.

S/N	Livestock/ livestock products	Number/ Amount	Total sold during last production season (no./ amount)	Price (Birr/ animals)	Total income (Birr)
1	Oxen				
2	Calf				
3	Cow				
4	Sheep				
5	Goat				
6	Poultry				
7	Camel				
8	Donkey				
9	Mule				
10	Horse				
12	Milk (lit)				
13	Butter (kg)				
14	Meat (kg)				
15	Hide & skin				
16	Egg				
17	Honey (kg)				
19	Others specify				

Expenditure for agricultural inputs

Expenditure listing	Amount (birr)
Improved seed	
Chemicals (fertilizer, insecticide, pesticide)	
Organic manure	
Hiring external labour	
Rent (land, machinery, other equipment)	
Transportation	
Livestock purchase	
Veterinary services	
Purchase/renting equipment	
Feed and water	
Fuel, maintenance and lubricants	
Tax, interest on loan etc	
Other inputs (specify)	

2.5. Has any member of your family or yourself worked on non-farm and off-farm activities? 1. Yes 2. No

2.6. Did you have access to extension services or advisory services? 1. Yes 2. No

2.7. Do you participate in the activities of farmers' training centers/pastoral training centers in your vicinity? 1. Yes 2. No

2.8.Mention your access to basic services (available in) your area

Access to basic facilities and services	Yes =1, NO=2
School (primary, secondary)	
Drinking water	
Electricity	
Human health center/pharmacy/clinic	
Livestock health center/veterinary services	
Market (daily, weekly)	
Farmer/pastoral training centers (FTCs/PTCs)	
College/university (private/public)	
Mobile coverage/telephone network	
Bank	
MFI (credit and saving)	
NGO office	

2.9.Are you a member of any social or community-based organization? 1. Yes 2. No

2.10. Do you have access to credit and saving organizations? 1. Yes 2. No

2.11. Participation in development interventions and your involvement

Development intervention	1. Yes 2. No	Your involvement/benefit
Youth, Women's and Children Affairs;		
Small and Medium Enterprise Promotion Office		
Productive Safety Net Program		
Irrigation cooperatives irrigation-based agricultural production		
Primary cooperative/cooperative union/farmer's organization		
Any other (please specify)		

2.33 Minimum dietary diversity (This section should preferably be conducted with a woman aged 15-49 years old. If there are no family members with such requirements, the survey may continue to be conducted with the family member who was already being interviewed. Select what you ate or drank in the last 24 hours. Please include all foods and drinks, any snacks or small meals, as well as any main meals. Remember to include all foods you may have eaten while preparing meals or preparing food for others)

No.	Food type	Examples	Yes, I ate	No, I did not eat

1	GRAINS, WHITE ROOTS and TUBERS	Corn/maize, rice, wheat, sorghum, millet , white potatoes, white yam, Enset, cassava, taro, sweet potato (white) or other foods made from grains and roots (e.g. bread, noodles, porridge, *injera* or other grain products)		
2	Pulses	Beans, peas, fresh or dried seed, lentils, fenugreek, chickpea, lupine, bean/pea products (eg. '*Shiro'*, '*hulbet'* etc)		
3	NUTS AND SEEDS	Groundnut/peanut, sesame, linseed, niger seed, rapeseed, sunflower or nut / seed "butters", oils or pastes		
4	DAIRY products	Milk, cheese, yoghurt, other milk products but NOT including butter, ice cream, cream or sour cream		
5	MEAT, POULTRY, FISH	Beef, lamb, goat, chicken, fish, seafood, animal organs (liver, kidney, heart or other organ meats or blood-based foods)		
6	EGGS	Eggs from poultry or any other bird		
7	DARK GREEN leafy VEGETABLES	Any medium to-dark green leafy vegetables, including wild / foraged leaves, spinach, cabbages, broccoli, etc		
8	DARK YELLOW or ORANGE FRUITS and VEGETABLES	Mango, papaya, pumpkin, carrots, squash, orange sweet potatoes, red pepper, lemon, watermelon, orange, mandarin, etc		
9	Other VEGETABLES	Cucumber, eggplant, mushroom, onion, tomato, etc.)		
10	Other FRUITS	Avocado, apple, pineapple, white sapote, banana, guava etc.		
11	Other	Specify any locally available food items (If any)		

2.34 Household dietary diversity score (HDDS) (food groups – score 0-12) + food consumption score (FCS)

No.	Food type	Examples	1. Yes<br>2. No	Freq. of consumption in the last 7 days
1	Cereals	Maize, rice, wheat, sorghum, millet, other foods made from grains and roots (e.g. bread, noodles, porridge, *injera* or other grain products)		
2	Roots or Tubers	White potatoes, white yam, enset, cassava, taro, sweet potato (white), orange sweet potatoes, etc.		

3	Vegetables	Cucumber, carrots, eggplant, pepper, mushroom, onion, tomato, dark green leafy vegetables, spinach, cabbages, broccoli, etc.)		
4	Fruits	Mango, papaya, pumpkin, squash, lemon, watermelon, orange, mandarin, avocado, apple, pineapple, white sapote, banana, guava etc		
5	Meat and poultry	Beef, lamb, goat, chicken, seafood, animal organs (liver, gizzard, kidney, heart or other organ meats or blood-based foods)		
6	Eggs	Eggs from poultry or any other bird		
7	Fish and Seafood	Fish		
8	Pulses/legumes/nuts	Beans, peas, fresh or dried seed, lentils, groundnut/peanut, fenugreek, chickpea, lupine, linseed, bean/pea/nuts products (eg. 'Shiro or stew', '*hulbet'*, nut butters, or pastes etc)		
9	Milk and milk products	Milk, cheese, yoghurt, other milk products		
10	Oils/fats	Vegetable oil, olive oil, animal fat (butter), fish oil, etc.		
11	Sugar/Honey	Honey, sugar, confectionaries, etc.		
12	Miscellaneous	Spices, red pepper, etc.		

Module 3. Occupational aspirations and barriers

3.5 Are you currently employed? Have you ever done any work for pay, do any kind of business, farming or other activity to generate income? (yes/no)

3.6 If yes, at what age did you start working/engaging in income generating activities?
If you are currently employed, which of the following best describes your employment status?

	Yes	No
Employed full time		
Employed part-time		
Multiple part-time jobs		
Self-employed (unpaid)		
Home-based work (paid)		
Others (specify)		

3.7 How would you rate your overall job satisfaction?

	Very satisfied	Satisfied	Neutral	Not satisfied	Not very satisfied
Current job satisfaction					

Was your current job/income generating activity your childhood dream? (yes/no)

If agriculture is not one of the sectors you are currently engaged in, can agriculture be a basic means of livelihood for female or male youth? (yes/no)					
	Strongly agree	Agree	Neutral	Disagree	Strongly disagree
When you compare agriculture and other professions, is agriculture a viable profession with a reasonable financial return? are youth able to support themselves and their families using agriculture?					
	Strongly agree	Agree	Neutral	Disagree	Strongly disagree

3.8 How would you rate the availability of appropriate employment opportunities for you?

	Very good	Good	Average	Not good	Very bad

Module 4. Evaluate existing policy strategies, practices, and development interventions in Ethiopia and identify factors that may enhance/constrain effective participation of rural youth in the study area

4.1 What policy strategies/practices/interventions/programs of government, non-government, civil society organizations (CBOs) and private sector are available in the area in the last five years to promote youth and women's engagement in programs, projects and interventions affecting their livelihoods?

List of policy strategies, practices, projects/interventions	Implementers (tick where relevant)				
	Public sector (government)	NGOs/international organizations	CBOs	Private actors	Others (specify)

4.2 Objectives, number of beneficiaries, target groups, target areas/Kebeles of the policy strategies, practices, and development interventions exist in the targeted areas that engage agro-pastoral youth to realize their potentials

List of policy	Objectives/goal	No. of	Target	Type of target

strategies, practices, projects/interventions	s/nature of support	beneficiaries (male/female)	Kebeles/Woredas	groups (who are the people these programs target?)

Module 5. 'Opportunity structures' in pastoral/agropastoral production system

a) What political, economic, social, technological and cultural structures exist to provide opportunities for youth engagement in pastoral/agropastoral system?

i. Available Opportunities for youth/women to influence pastoral/agropastoral policies among these people or institutions? [Including holding leadership positions in these groups; participate in decision – making

	Who or which institutions facilitate these opportunities	How often do you participate in activities and meetings (decision-making and meetings organized by these groups?			
	Yes=1<br>No=2	Never/Almost never	Always	Sometimes	Most of the time
Community welfare group					
Women's group					
Youth Group					
Men group					
Cooperatives/Union					
Religious groups					
Farmers field school					
NGO's					
Donor organization					
Research Organization					
Private Organization					
Others					

ii. Are they viable platforms to improve women and youth engagement in agriculture? Yes No

Module 6. Design, implement and evaluate demand-driven capacity-building interventions that may enhance rural youth's entrepreneurial skills and capabilities, livelihood, and empowerment. … Training needs assessment questions

6.1 Did you obtain training over the last five years? 1. Yes 2. No

6.2 Would you be interested in starting your own business? Yes/no
